# Family graveyards form underappreciated local plant diversity hotspots in China's agricultural landscapes

**DOI:** 10.1038/s41598-020-80362-6

**Published:** 2021-01-21

**Authors:** Cheng Gong, Liangtao Li, Jan C. Axmarcher, Zhenrong Yu, Yunhui Liu

**Affiliations:** 1 College of Agricultural Resources and Environmental Sciences, China Agricultural University, Beijing, 100193 China; 2 College of Landscape and Ecological Engineering, Hebei University of Engineering, Handan, 056000 Hebei China; 3 UCL Department of Geography, University College London, London, UK; 4 Faculty of Environmental and Forest Sciences, Agricultural University of Iceland, Borgarnes, Iceland

**Keywords:** Agroecology, Biodiversity, Conservation biology, Ecosystem services

## Abstract

In the intensively farmed, homogenous agricultural landscape of the North China Plain, family graveyards form distinct cultural landscape features. In addition to their cultural value, these graveyards represent semi-natural habitat islands whose potential roles in biodiversity conservation and ecological functioning has remained poorly understood. In this study, we investigated plant species richness on 199 family graveyards of different ages and sizes. In accordance with biogeography theory, both overall and insect-pollinated plant species richness increased with area and age of graveyards. Even small graveyards show a strong potential for conserving local plant richness, and a mosaic of both large and small family graveyards could play an important role in the conservation of farmland biodiversity and related ecosystem functions. The launch of agri-environmental measures that conserve and create semi-natural habitats, in turn benefitting agricultural biodiversity and ecological functioning, has proven difficult in China due to the shortage of dispensable arable land. Given the great value of family graveyards as semi-natural habitats reflected in our study, we propose to focus preliminary efforts on conserving these landscape features as existing, widespread and culturally important semi-natural habitat islands. This would represent an effective, complementary policy to a subsequent re-establishment of other semi-natural habitats for the conservation of biodiversity and ecological functioning in agricultural landscapes.

## Introduction

The impact of biodiversity losses on the primary productivity of ecosystems and on ecosystem functioning is well documented^1–3^. In agricultural landscapes, these losses commonly trigger a decline of various ecosystem services^4,5^, with habitat losses from land use change, particularly related to the homogenization of agricultural landscapes, and related stresses identified as major threats to biodiversity^6–8^. Semi-natural habitats that are known to support biodiversity across various important functional groups of organisms, ranging from plants and insect pollinators to predatory arthropods^9–11^, have widely disappeared from modern agricultural landscapes. These habitats have been reported to positively impact on a range of ecosystem services in agricultural landscapes^5,9,12^. In widely homogenized agricultural landscapes, even small remnant semi-natural habitat islands appear to have a noticeable positive effect on biodiversity and ecosystem services^13^. In Europe, Agri-Environment Schemes (AES) aimed at reversing losses in agricultural biodiversity and ecosystem services therefore have made the conservation and creation of semi-natural habitats in agricultural landscapes a key strategy^14^.

In the North China Plain, little natural and semi-natural habitat remains due to the long history of agricultural land use and the ever-increasing pressure to feed a large and growing population. One semi-natural landscape feature with regional cultural characteristics that has remained relatively common are family graveyards. According to funeral traditions developed in the last century, farmers bury dead family members directly on their arable land. After establishment, these graveyards are rarely interfered with, and not used for crop cultivation. Over time, graveyards have expanded and can now be seen as a patchwork of semi-natural habitat islands in the intensively used agricultural landscape matrix.

Island biogeography theory (hereafter IBT) has formed the foundation of studies into factors influencing species richness on islands^12^, and as a key explanatory theory for diversity patterns in plants^15^. Family graveyards are normally circular in shape and show spatio-temporal variations in relation to the number of buried family members. According to IBT, research on terrestrial habitat fragmentation^16,17^ has commonly reported for natural or semi-natural habitat fragments in anthropogenically transformed landscapes to act analogous to “proper” islands. Islands generally harbor an equilibrium numbers of species, and this number is determined by island size and isolation, with greater size and smaller distances to similar types of habitat increasing diversity^18,19^. While there is a strong and growing body of literature assessing the general conservation value of semi-natural habitats in agricultural landscapes both globally and in China, the value and behavior specifically of Chinese family graveyards in this context received very limited attention^20^.

Despite their cultural importance, family graveyards are also increasingly threatened by land use change. The rapid urbanization in China has triggered local losses of arable land and, crucially, rural labor, in turn leading to a series of land consolidation projects (LCPs) launched by the government that aim to create large homogenous arable land areas to facilitate mechanization and increase the overall area of arable land. The goal of these projects is to ensure food security by keeping the total arable land area above a ‘Red Line’ of 120 m ha. The resulting agriculture-focused LCPs in China have led to increases in arable land—at the cost of non-cropped habitats and biodiversity-associated ecosystem services^21^. LCPs require lost arable land to be replaced with the same size of newly reclaimed land, and as a result, the use of good arable land as new family graveyard is prohibited throughout most of China. Despite some objections from local populations, existing graveyards are also facing threats of reconversion to arable land to promote an intensive, highly mechanized agricultural production. This is leading to accelerating destructions of family graveyards and associated biodiversity losses. In turn, these developments have exacerbated reductions of multiple ecosystem services that are important in supporting sustainable agricultural production and rural development.

To evaluate the role of graveyards as semi-natural islands in biodiversity conservation, we targeted plant diversity. Plants are key ecosystem components as primary producers in terrestrial ecosystems^22^ that can influence the community structure across trophic levels by providing food resources and habitat structure^23–25^. Plant species diversity is important for preserving the productivity^26–28^ and stability^29,30^ of ecosystems. In addition to overall phylodiversity, we paid particular attention to the species richness of insect-pollinated plant species. These plants provide essential sustainable food resources for many pollinator species in an otherwise resource-poor landscape, allowing them to provide critical pollination services in the short time-frame where it is needed for the sustainable production of insect-pollinated crops^31,32^. Earlier studies have clearly shown that pollinator diversity is enhanced by high levels of plant species richness^33^, as well as by the abundance of nectar-providing plants^34,35^. We therefore believe that studying insect-pollinated plants in family graveyards can reflect the contribution these cultural landscape features can make to enhance local pollinator assemblages and associated pollination services.

In our study, we specifically test the hypotheses that: (I) in comparison with other local habitats, family graveyards provide an important refuge for species-rich plant assemblages; (II) the richness of plants overall and of insect-pollinated plants increases with the increase in area and age of graveyards; and (III) even small graveyards contain high levels of plant diversity. We discuss implication of our findings for land use management and policy making.

## Results

We recorded a total of 81 plant species at the 199 family graveyards, representing 70 genera and 30 families. Eight plant species were shrubs or small trees (height < 2 m), while the remaining 73 species were herbs. The average species richness/graveyard was 14.45 (SD: 6.02). The smallest family graveyards covering an area of merely 2 m^2^ already contained 12 plant species. The accumulated diversity encountered on quadrats representing the 24 smallest graveyards (each < 10 m^2^) of 43 plant species already accounts for 53% of all plants recorded on the graveyards.

**Figure 1:**
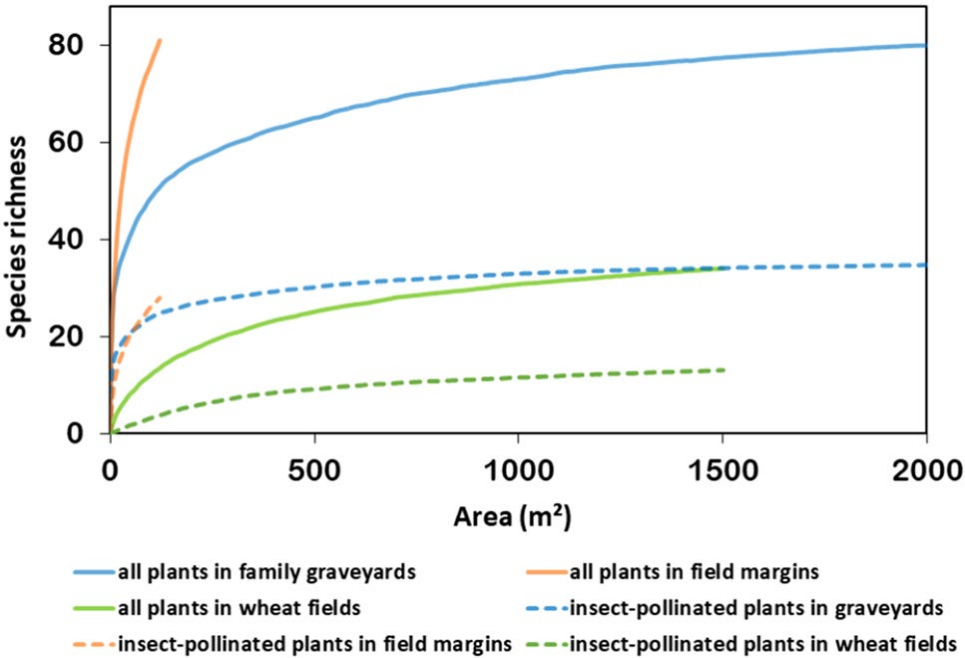
Area-based rarefaction curves comparing plant species richness of the 3 different habitat types. Continuous and dashed lines represent the numbers of ‘overall plants’ and ‘insect-pollinated plants’, respectively.

A total of 81 plant species belonging to 27 families was also recorded in surveys of the 30 regional field margins (average 19.03 ± 4.69 species/margin), although in this case, the overall surveyed area (i.e. the total area covered by survey quadrats) was significantly smaller, and rarefaction curves (Fig. 1) indicate that the species number has clearly not reached a saturation point. Surveys of the 125 sites within wheat fields in contrast only yielded a total of 34 plant species belonging to 14 families (average 3.42 ± 1.23 species/site). The combined records for all habitats included a total of 114 plant species, and only 29 species occurred in all three habitat types. Overall, the average plant species density (species/m^2^) was lower in graveyards than at field margins in the same county, but significantly higher than in the surrounding arable land.

A total of 35 plant species were categorized as insect-pollinated (average 7.43 ± 3.96 species/graveyard), accounting for 43% of the total vascular species pool, and belonging to 33 genera and 18 families. *Asteraceae* (10 species) account for 55% of all insect-pollinated plant records, making this family the most important insect-pollinated taxon at graveyard sites. In comparison, 28 insect-pollinated plants (average 5.70 ± 2.56 species/margin) were recorded at field margins, accounting for 35% of the overall plant species pool at the field margins. Only 13 insect-pollinated plant species were found in wheat fields (average 1.48 ± 0.78 species/site). The rarefaction curves for insect-pollinated plants in family graveyards (Fig. 1) again indicate a similar richness to field margins.

**Figure 2:**
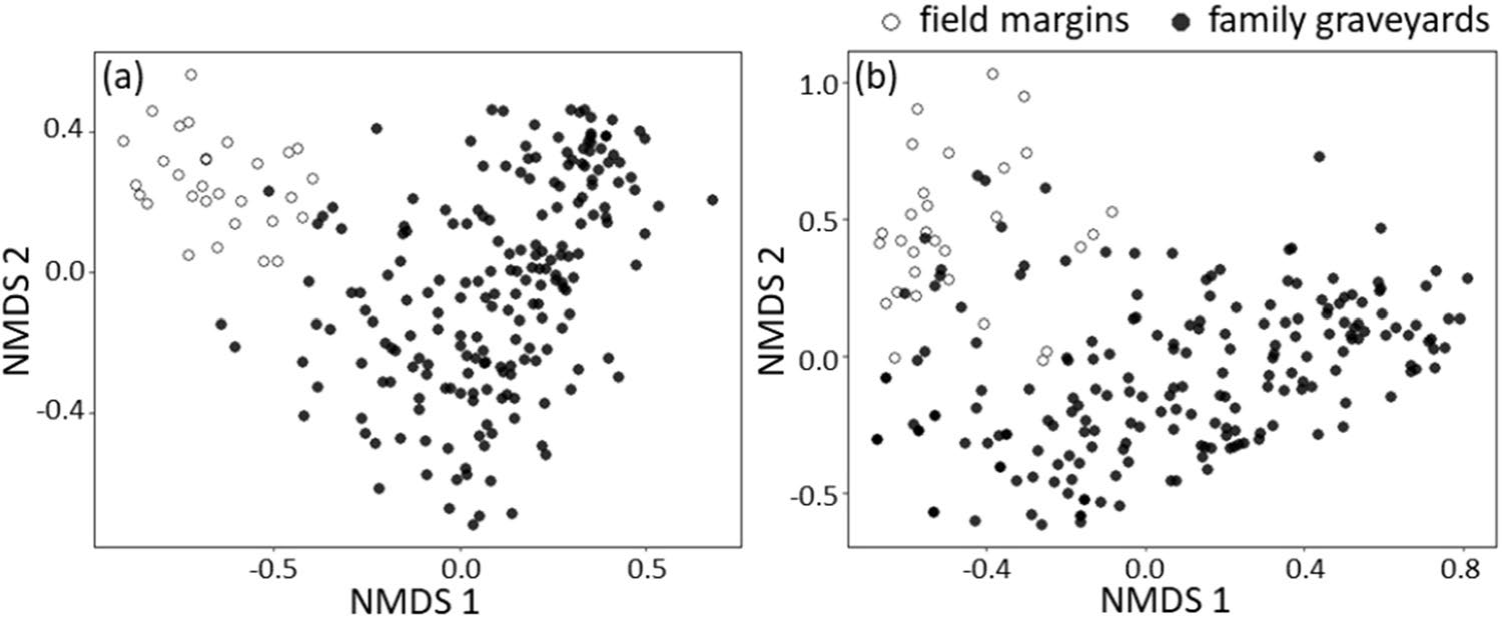
NMDS ordination plots based on the Bray–Curtis dissimilarity matrix reflecting (**a**) the overall plant species composition and (**b**) the insect-pollinated plant species composition at the sampling plots of two main habitat types—field margins and graveyards.

When comparing the vegetation composition, field margins and family graveyards varied strongly, although our records for both habitat types included an identical number of 81 plant species. Only 48 of these species were shared between the two habitats, while the remaining 66 plant species were unique to records from either of the two habitat types. This is also reflected by the two distinct clusters that show only limited overlap in the NMDS ordination plot (Fig. 2a—stress = 0.004, linear fit: r^2^ = 0.99). This trend was replicated when focusing uniquely on the assemblages of insect-pollinated plant species, only, although the NMDS ordination plot (Fig. 2b—stress = 0.005, linear fit: r^2^ = 0.99) shows slightly more overlap between the two habitat types. Overall, the field margins shared 16 insect-pollinated species with the family graveyards, while the remaining 30 insect-pollinated plants were recorded from either of two different semi-natural habitat.

Family graveyard size varied between 2 and 400 m^2^, and their total area amounted to 10,865 m^2^, with an average size of 55 (± 62.57) m^2^. While arable land in the study area covered on average 8,142 m^2^/ha, family graveyards only occupied 10 m^2^/ha, or 1 ‰. The oldest graveyard has been used for six generations, with the oldest burial sites therefore dating back ~ 180 years. The average age of family graveyards surveyed was 2.55 (± 1.39) generations.

**Figure 3:**
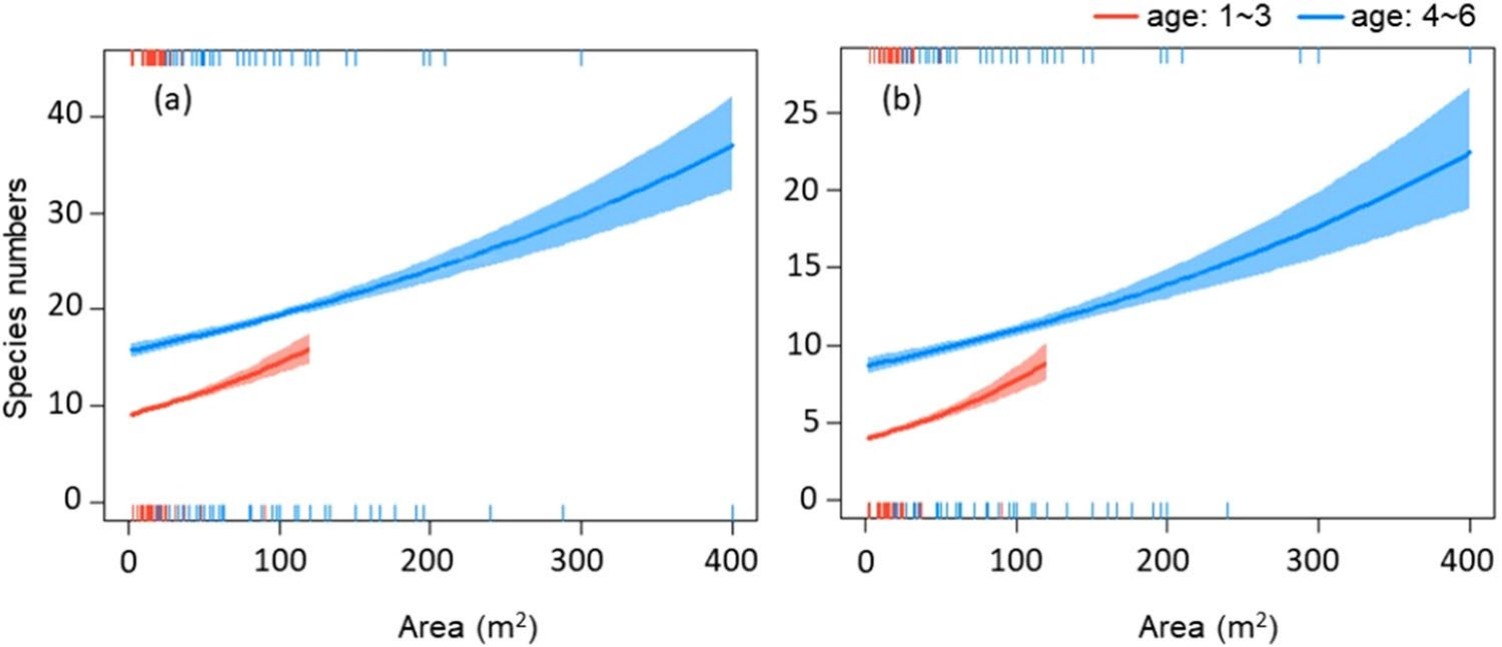
Effects of the interaction between area and age on species numbers of all plants (**a**) and insect-pollinated plants (**b**). The red line shows the effect trend of area on plant species numbers in young graveyards (< 4 generations), while the blue line represents the trends for older graveyards. The colored areas reflect the 95% confidence intervals for the two models.

Plant species numbers were found to be positively linked to both, graveyard area and age (*p* < 0.01, Fig. 3a), with these trends reflected also in the insect-pollinated plant species numbers (*p* < 0.01, Fig. 3b). General linear models furthermore predicted differential effects of area on the species numbers, with stronger effects observed in young family graveyards (< 4 generations) when compared to older ones.

## Discussion

In China, strong cultural value systems and emotional connections generated by kinship relationships stretching across generations have influenced a wide range of traditional burial customs. Only relatives of the graveyard owners may enter these sites on selected days, allowing family graveyards to be protected from human interference for most of the year, with graveyards experiencing natural succession and vegetation restoration. Compared with the intensive arable land around them, these semi-natural habitats therefore represent local plant diversity hotspots in the arable land. While their phytodiversity levels were not dissimilar to semi-natural field margins, the relatively low level of overlap in species between field margins and family graveyards indicates a unique role of graveyards in preserving local plant diversity. Our results therefore confirm the great potential value of family graveyards for conservation efforts in agricultural landscapes. In line with earlier studies for other semi-natural habitat types^18,36,37^, our results reflected a strong positive relationship between the size of a family graveyard and the plant richness contained in our vegetation samples. This is likely linked to larger habitat patches containing a greater heterogeneity of environmental conditions like microclimatic and soil conditions, as well as to their ability to sustain larger species’ populations, in turn reducing extinction risks of these species that favors a greater diversity^38^. Furthermore, larger fragments tend to have longer perimeters that can also better support potential specific ecotone species and assemblages^36^, and they have a higher chance for successful colonization by new species since they are more easy to detect within the landscape matrix, while also providing more stable environmental conditions in their central areas when compared to smaller fragments^39^.

The size of a Chinese family graveyard is generally correlated with their age. According to local funeral customs, graveyards belong to families, not individuals. Over time, more family members will hence be buried at the site, automatically leading in an increase in the required area. Nonetheless, while this increase in size is generally linked to an increase in plant species richness, this relationship appears to weaken over time. This might reflect the tendency that over longer periods of time, more and later-succession plant species colonize, outcompeting early pioneer species with graveyard assemblages approaching a saturation point that is likely more strongly determined by the overall local species pool than by their actual area, with graveyard species composition homogenizing over time^40^. In an ideal scenario, we therefore should preserve arrays of graveyards of both different ages and sizes, with size variations particularly important in young graveyards.

While our survey plots on large graveyards harbored a particularly high diversity of plant species, even small family graveyards appear to play important roles in conserving the plant species richness in the agricultural land-use matrix. The potential importance of small habitat patches for biodiversity conservation is well established^10,13,41,42^. Even the smallest graveyard (2 m^2^) with its 12 plant species already represents a distinct diversity ‘hotspot’ in the highly homogenous intensively cultivated agricultural landscape. Since graveyard ages, environmental settings, land-use histories and local management structures are variable between individual patches, vegetation assemblages representing discontinuous small patches are instead likely to be highly variable. Furthermore, the crucial role of graveyard age for biodiversity particularly in small graveyards highlighted by our models likely reflects the enhanced opportunity of plant species to both colonize and subsequently establish sustainable populations at these small graveyards afforded by longer timeframes.

Our analysis of insect-pollinated plants is highly consistent with that of all plants, with model results showing minimal variations. Graveyard, as a type of semi-natural habitat, support a higher level of species numbers of insect-pollinated plant while occupying less land than field margins. Furthermore, the most common semi-natural habitat types in the intensively cultivated agricultural landscape of the North China Plain are planted windbreaks and other woodland that result in heavily shaded habitats presenting very limited resources for insect pollinators^43^, while their homogenous vegetation structure and frequent disturbance furthermore limit their value as local biodiversity hotspots^44,45^. Our study therefore indicates that the protection of graveyards could play a crucial role specifically in conserving and promoting insect-pollinated plants, potentially greatly benefitting pollination services and also biological control services associated with beneficial insects that depend on open, phytodiverse habitat types.

Family graveyards are however disappearing rapidly with current changes of land-use and the implementation of current LCPs in rural areas. Since the late 1990s, establishing new graveyards has been banned in most regions of China to prevent the growing number of graveyards from taking up more arable land. Even many existing family graveyards have already become reclaimed for farming to satisfy the demand of new arable land area. Our study gives reason to pause this extermination of family graveyards, emphasizing their valuable role as semi-natural habitats for biodiversity conservation.

In China, a coherent compensation policy for conservation or establishment of semi-natural habitats in arable landscapes is still lacking, and a development of policies similar to AES will require substantial time. Even if sound financial compensations were available, the application of AES in Europe is known to be strongly determined by farmers’ uptake and acceptance of individual components of these schemes, which are linked to utilitarian motivations such as payment rates and ease of fit within existing farming practice^43,46^. Family graveyards use only a very small amount of land resources to harbour high levels of phytodiversity, with little additional cost. Instead of protecting and managing other semi-natural habitat such as field margins and road verges, farmers are generally more willing to participate in the preservation of family graveyards, driven not least by both cultural reasons and the farmers’ emotional connections.

Overall, the conservation of family graveyards represents a highly acceptable approach for farmers to protect and further extend semi-natural habitats—even where there is no subsidy available. We are convinced that more consideration should therefore be given specifically to the conservation of family graveyards within China's ecological protection policies.

We therefore call for LCPs to specifically encourage the preservation of existing family graveyards—regardless of their size and age, as highly effective complementary, or even alternative components of agri-environmental measures and land consolidation processes to enhance biodiversity and its service provision to agricultural landscapes. In the future, with the promotion of cremation and further declines in the rural population, the area of family graveyards is likely to remain widely constant at best, and policies for the conservation of graveyards do not entail the risk of taking up rapidly expanding, new areas of arable land. These family graveyards will in turn become historical relics of traditional funeral culture, as well as aligning with goals to increase sustainable agriculture and the quality of the environment in general. Since there is no need to build and manage new areas in this context, family graveyards are seen as a key asset in emerging strategies aimed at enhancing the sustainability of agricultural production via the promotion of diversity-related ecosystem services—with little cost to the government. While in the longer term, tailored landscape design incorporating the establishment of additional semi-natural landscape elements will be required to further increase agricultural sustainability^47,48^, we believe that graveyards can make a small, but important contribution towards this process.

## Methods

### Study area

This study was conducted in Quzhou county (36°36 N, 114°50E) in the south of Hebei Province in a representative agricultural landscape for the main cereal production area of the North China Plain. The average temperature at the study region is 14.1 °C (range 13.0–15.4 °C), and the average annual precipitation reaches ~ 438 mm (range 219–792 mm; National Meteorological Bureau, period 1994–2014). The elevation varies from 32.7 to 45.4 m above sea level.

Characterized by intensive agriculture production and with a mosaic of small fields dominating the landscape, the study area has a long tradition of crop planting, interspersed with few linear semi-natural habitat structures like windbreaks and field margins, and some small semi-natural islands like family graveyards. In the past decades, the land in the region was intensively managed. The local arable land is cultivated by rotations of summer maize and winter wheat, resulting in a highly intensive and homogenized agricultural landscape. According to statistical data from 2012, the county was principally covered by agricultural land (81% of the land area), followed by built land (6%), water (4%), and transport infrastructures (3%), with very small proportions remaining for woodland (2%) and orchards (less than 0.1%).

Family graveyards are common in the study area and across many other rural areas in northern China. Usually, a graveyard is created on a local family field following the death of a family member. Being near-circular when first established, traditional graveyards contain a small area for the tombstone and are otherwise covered in local native vegetation. As time goes by, more family members are buried on the graveyards which take on an increasingly area. Each graveyard generally belongs to an individual local family and established directly on the arable land cultivated by that family. In consequence, family graveyards generally form isolated islands within the agricultural farmland.

### Field survey

**Figure 4:**
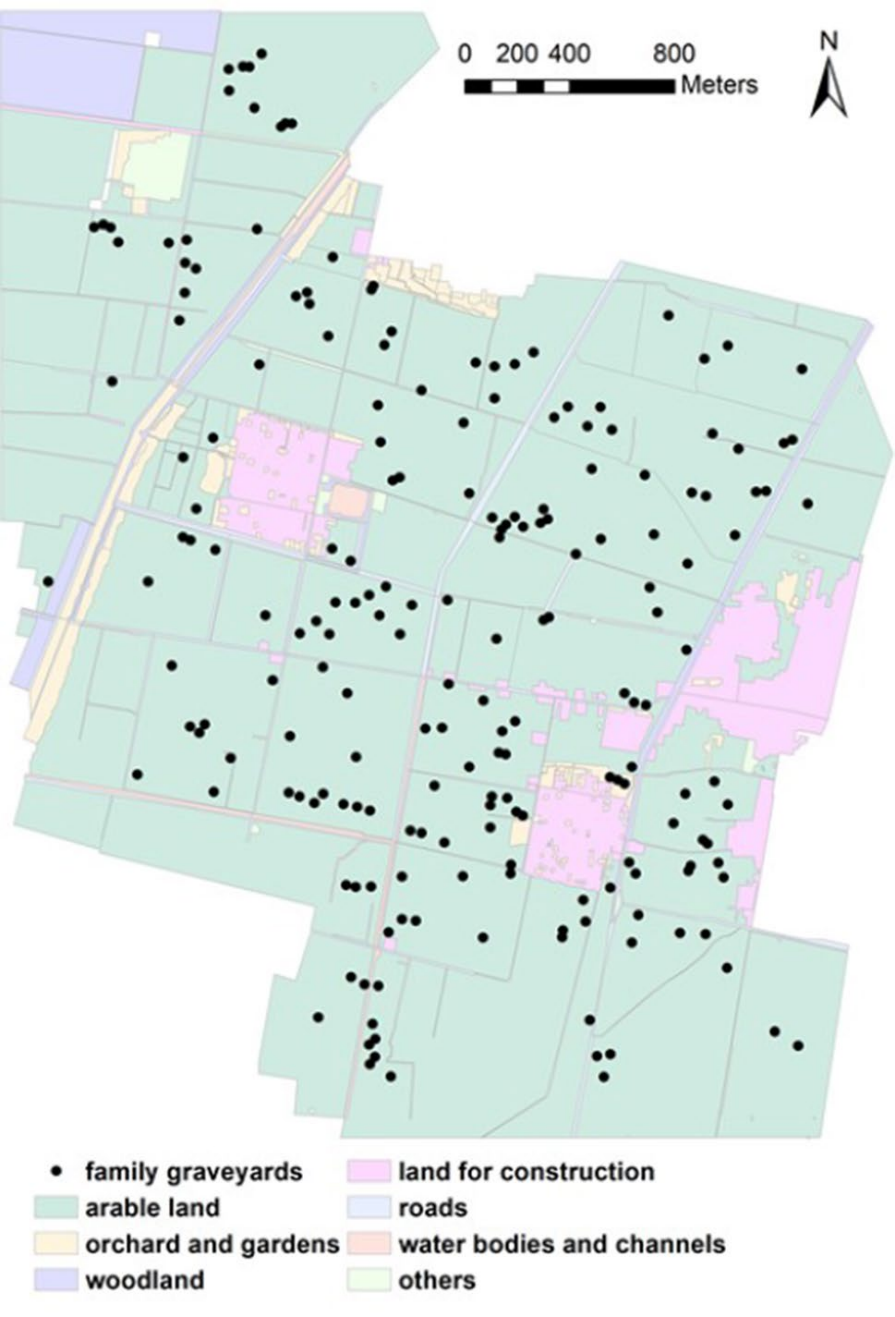
Location of the 199 family graveyards on arable land. ArcGIS10.2^49^ was used to create this map. The data of land use and family graveyards location comes from the field survey by the second author of this article.

In 2012, a total of 199 family graveyards ranging in size from 2 to 400 m^2^ were selected for plant surveys (Fig. 4). The arable land around the family graveyards in our study area is generally flat and homogeneous. Plants were surveyed across all graveyards in June and September, and all species encountered during the two surveys were recorded for each graveyard. The area of each graveyard was accurately measured during the plant surveys. Since no trees with a height > 2 m were found in any survey, the vegetation of each graveyard was stratified into woody shrubs/small trees and herbs.

Within the footprint of each family graveyard, we randomly selected three plots of 2 m × 2 m for detailed plant surveys. We adjusted the spread of these plots according to the shape of family graveyard, while the area remained unchanged. All plant species were recorded on the plots and summed to create the respective species richness. For graveyards < 4 m^2^, all plant species were recorded on the entire graveyard. The same observer carried out the sampling at all sites. In carrying out the plant survey, we measured the area of each family graveyard on site using a GPS. Since the family graveyard is surrounded by uniform and intensively cultivated farmland, its boundaries are obvious. The age of each family graveyard was obtained by interviewing local farmers, specifically members of the family who own the graveyard land. Where families keep family trees (genealogical record), we checked this information to determine the history and age of the graveyard. For some graveyards, if no descendants recently resided in the local villages, we enquired the local elders who had lived in the village for > 60 years and those who have presided over or attended funerals at the respective graveyards. According to the burial customs and descriptions of the locals, the history of the general family graveyard is recorded on a generational basis and can be compared with the family tree. We coded graveyard ages in terms of intergenerational relationships, with an inter-generational time-span of 30 years used as basis for these calculations.

To compare the plant species in family graveyards with that on the surrounding arable land and field margins as another semi-natural habitat type, we surveyed plants in intensive wheat fields and surveyed plant species at field margins within the same county. We randomly selected 125 points in wheat fields within this landscape (each point > 10 m from the nearest field margin) as centers of circles with a radius of 5 m, positioning three plots of 2 m × 2 m within each circle. All plants with each plot were recorded. The data of plant species numbers in the field margins were obtained from an experiment organized by the corresponding author in the same county in May and September of 2014^50^. Here, all tree and shrub species were recorded from 30 field margins, with herbaceous plants recorded in four 1 m^2^ plots randomly positioned along the length of each field margin in a way that maximized distances to the neighboring fields.

### Data analysis

We standardized the data using rarefaction curves for the purpose of direct comparison of the plant species richness for a standardizes sample area size since the overall sampling efforts varied between different study regions^51^. The area-based rarefaction is a proven method that provides support for standardized comparisons of samples generated through different sampling efforts (here size of sampled area)^52^. We used area-based rarefaction for plants rather than sample-based (numbers of sites) because different numbers of plots and different plot sizes were used in the different surveyed habitats. In this context, area‐based rarefaction for incidence data was selected since it takes full account of the differences in the overall survey areas sampled for the different habitats.

To visualize the resulting dissimilarity matrices for both, the overall vegetation and insect-pollinated plant species, only, between sites recorded on field margins and family graveyards, we performed a non-metric multidimensional scaling (NMDS) using the vegan (version 2.5-6)^53^ software package in R. As a distance measure, the Bray–Curtis dissimilarity calculations based on presence-absence data was used, which is one of the most robust measures for this purpose^54^.

The responses of species numbers to area and age in family graveyards were computed using general linear models (GLM) assuming a Poisson distribution (package “MASS”, version 7.3–51.5)^55^. Due to the correlation between area and age (r = 0.79), but with limited collinearity between them detected based on variance inflation factors (VIF = 2.62)^56^, interactions between the area and age of family graveyard were included as explanatory variables in the model, with species numbers (sum of plant species observed per 3 plots) of overall and insect-pollinated plants included as response variables, respectively. We tested the significance using Z-tests, with a *p*-value < 0.05 indicating a significant effect ^55^.

Spatial autocorrelation was assessed using Moran's I values based on geographic coordinates and the species richness variables using the package ‘spdep’ (version 0.7–7)^57^, and a significant spatial autocorrelation was detected (*p*-value < 0.01 in the case of overall plants and insect-pollinated plants). We therefore used the Moran eigenvector filtering function intended to remove spatial autocorrelation from the residuals of generalised linear models. It uses ‘brute force eigenvector selection’ to reach a subset of optimized vectors to be included in the GLM. Finally, we validated the models based on visual inspection of the plotted residuals versus the predicted values. All the above-mentioned analyses were performed in R version 4.2.0^58^.

In parallel, we analyzed and determined insect-pollinated plant species richness from our surveys, based on literature of insect-pollinated plant species^59–62^. We firstly gave a score from 0–6 to each plant species according to the shape and quantity of their flowers, the amount of pollen and nectar presented, as well as the preferences of insect pollinators. Zero indicates that a plant species has no supporting pollinator, and 6 indicates that a plant’s flowers strongly support insect pollinators. We then categorized all plant species with ≥ 3 points as insect-pollinated plants and re-ran our analysis for insect-pollinated plants, only.
